# Regional Processes Mediate Ecological Selection and the Distribution of Plant Diversity Across Scales

**DOI:** 10.1111/ele.70095

**Published:** 2025-03-05

**Authors:** Christopher P. Catano, Jonathan Bauer, Tyler Bassett, Eric Behrens, Lars A. Brudvig

**Affiliations:** ^1^ Department of Botany & Plant Sciences University of California Riverside California USA; ^2^ Department of Plant Biology Michigan State University East Lansing Michigan USA; ^3^ Program in Ecology, Evolution, & Behavior Michigan State University East Lansing Michigan USA; ^4^ Department of Biology Miami University Oxford Ohio USA; ^5^ Institute for the Environment and Sustainability, Miami University Oxford Ohio USA; ^6^ Michigan Natural Features Inventory Lansing Michigan USA; ^7^ National Park Service Southern Plains/Rio Grande Fire Groups USA

**Keywords:** alpha & beta diversity, community assembly, determinism versus stochastic drift, grassland ecosystem restoration, immigration, landscape experiment, species pools, species sorting

## Abstract

Community ecology remains focused on interactions at small scales, which limits causal understanding of how regional and local processes interact to mediate biodiversity changes. We hypothesise that species pool size and immigration are two regional processes altering the balance between local niche selection and drift that cause variation in plant diversity. We manipulated the richness and number of seeds sown (species pool size and immigration respectively) into 12 grasslands across a landscape soil moisture gradient. Greater immigration and smaller species pools increased the variation in plant composition explained by soil moisture gradients but resulted in greater erosion of plant *α*‐diversity and spatial *β*‐diversity over time. Our results suggest that regional constraints on colonisation make community assembly more variable but help maintain species diversity by limiting biotic homogenisation. This study provides large‐scale experimental evidence on how regional contexts can alter the relative importance of fundamental processes shaping biodiversity change across scales.

## Graphical abstract

This experiment quantifies how species pool size and immigration influence plant community assembly in restored grasslands. Our findings reveal that greater immigration (seed arrival) and smaller species pools (fewer arriving species) promote niche selection over ecological drift, driven by stronger species sorting across a landscape soil resource gradient. This study provides large‐scale experimental evidence on how regional contexts can alter the relative importance of fundamental processes shaping biodiversity change across scales.

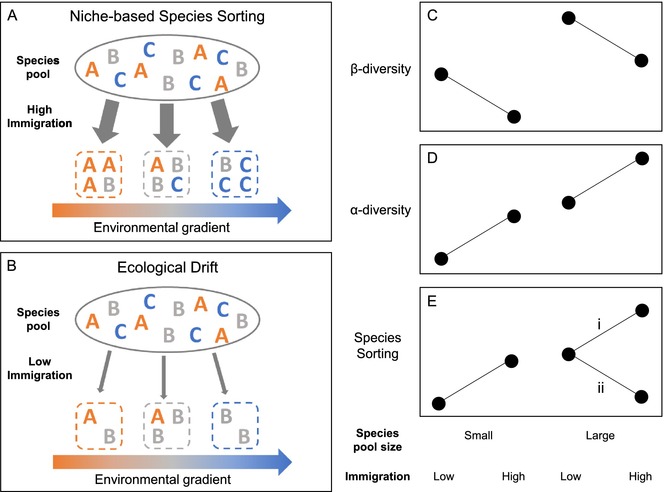

## 1 Introduction

Biologists have long recognised that biodiversity patterns depend on processes operating at different scales (Leibold et al. 2004; Levin 1992; Wiens 1989). Yet local perspectives continue to dominate, whereby species interactions within local communities are thought to underpin biodiversity patterns (Chesson 2000; HilleRisLambers et al. 2012; LaManna et al. 2017). Challenging this view, regional perspectives suggest macroevolution and large‐scale immigration are more important for structuring biodiversity within and among local communities (Harrison and Cornell 2008; MacArthur and Wilson 1967; Ricklefs 2008). However, debates regarding regional versus local constraints on biodiversity (Harmon and Harrison 2015; Rabosky and Hurlbert 2015) can dichotomise processes that are linked across scales by dispersal (Leibold et al. 2004; Leibold and Chase 2017). Moreover, neither perspective has resolved one of the biggest challenges at the interface of ecological theory, global change biology and biodiversity conservation: why does biodiversity change often appear variable and challenging to predict (Lawton 1999)? While biodiversity change can be generalised as the product of fundamental ecological processes—dispersal (immigration and movement of individuals), ecological drift (changes in relative abundance that are random with respect to species' identities) and niche selection (changes that are deterministic due to species' fitness differences) (Vellend 2010, 2016)—it remains unclear the extent to which regional and local contexts interact to shape the relative importance of these processes.

**FIGURE 1:**
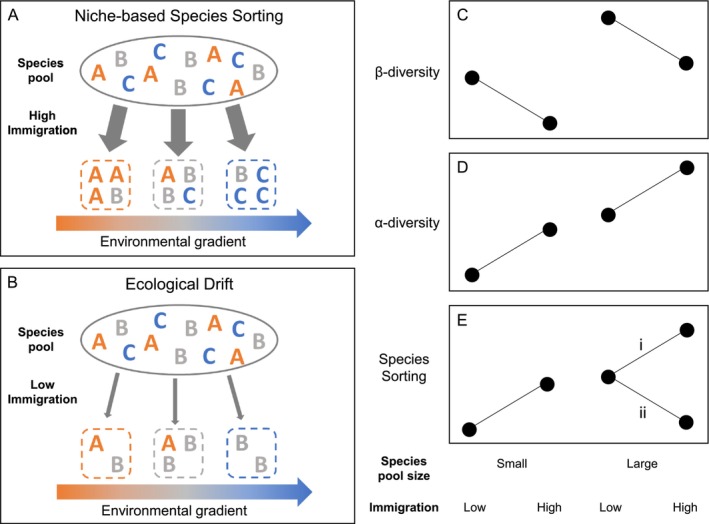
Influence of species pool size and immigration on community assembly that results from processes of (A) niche‐based species sorting and (B) ecological drift and predicted diversity patterns (C–E). Species are represented by letters and colours correspond to the environmental conditions where their fitness is maximised; squares are local communities along an environmental gradient. Predicted effects of species pool size and immigration on assembly outcomes: (C) spatial variation in community composition (*β*‐diversity), (D) local diversity (*α*‐diversity) and (E) the relationship between compositional variation and differences in environmental conditions. A greater or smaller percentage of compositional variation explained by the environment indicates stronger (i) and weaker (ii) species sorting respectively. Our experiment manipulates species pool size (2 levels: 8 vs. 30 species) crossed with immigration (4 levels: 270, 485, 700, 970 seeds m^−2^) in 12 sites spanning a landscape soil moisture gradient.

We hypothesise that two regional processes—immigration of individuals and species pool size (Figure 1)—alter the balance between niche selection and drift that cause variation in plant composition. Immigration could increase the relative importance of selection through two, nonmutually exclusive mechanisms. First, higher immigration can overcome chance colonisation events that make community assembly less predictable. For example, fewer arriving individuals can limit the likelihood that the superior competitors in a region colonise local communities, thereby allowing more subordinate species to establish (Hurtt and Pacala 1995). Second, higher immigration can promote deterministic outcomes of competition by increasing the number of individuals in a community (i.e., the effective community size) (Orrock and Watling 2010; Ron et al. 2018; Vellend et al. 2014).

**FIGURE 2:**
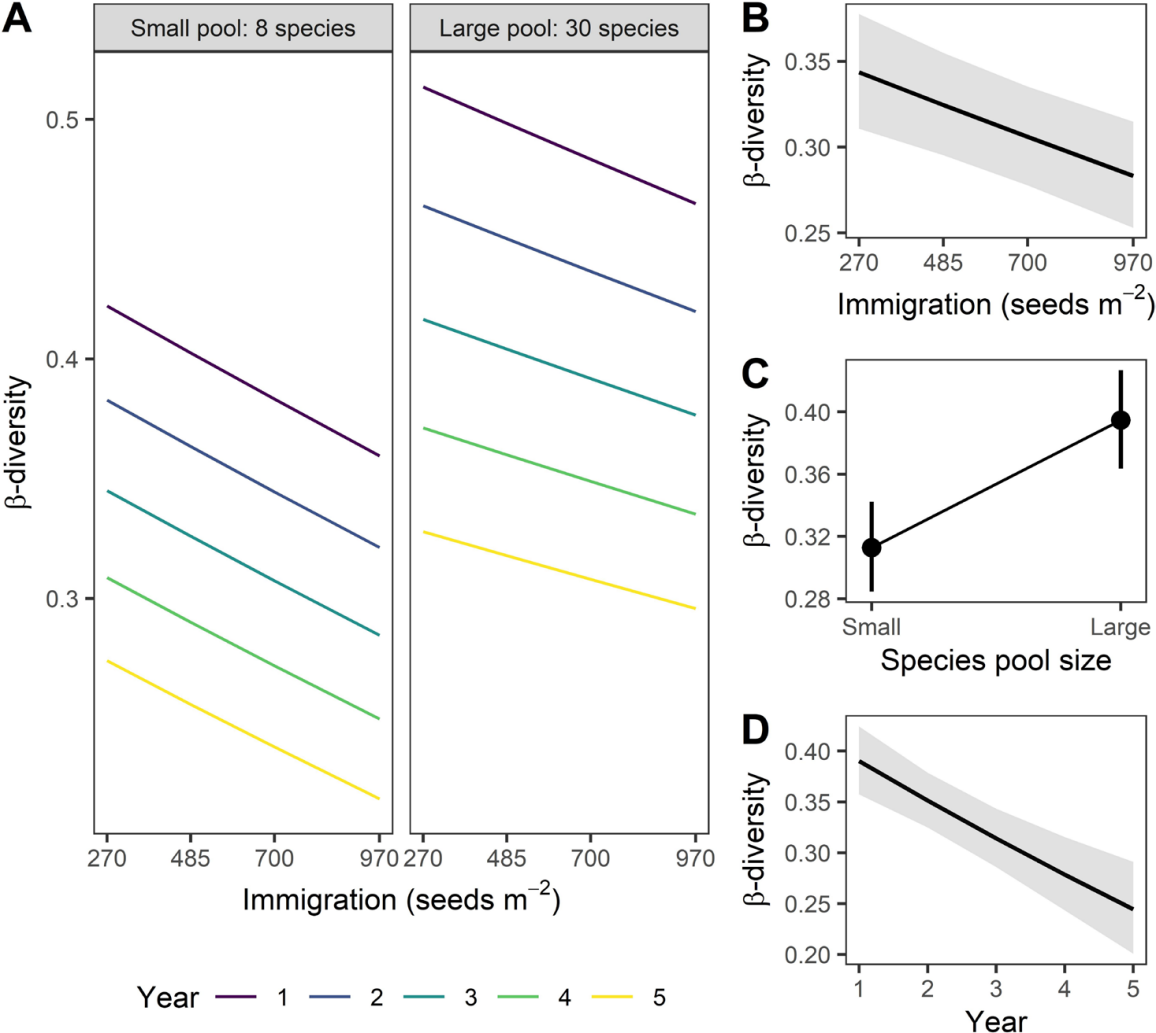
Effects of Immigration, Species pool size and Time (Year) on variation in plant community composition (*β*‐diversity; distance‐to‐centroid using Bray–Curtis dissimilarity). There was no clear evidence for interactions between any input variables (A). Immigration (B), Species pool size (C) and Year (D) are the model predictions (marginal effects) conditioned on the other input variables fixed at their mean, with 95% CIs. All main effects are statistically significant (Table S2). The model was fitted to square root transformed *β*‐diversity, with random slopes for years (*n* = 5) and random intercepts for sites (*n* = 12), and results are plotted on the scale of the original untransformed data (*n* = 2606). Model predictions and data for each site are shown in Figure S3.

The hypothesis that immigration increases selection relative to drift (Figure 1A,B) makes three key predictions: (1) higher immigration decreases variation in species composition across local communities (spatial *β*‐diversity; Figure 1C), (2) increases species diversity within local communities (*α*‐diversity; Figure 1D) by mitigating probabilistic extinctions and (3) strengthens species‐environment relationships (species sorting; Figure 1E) (Mouquet and Loreau 2003). Despite immigration being central to biodiversity theories (Leibold and Chase 2017; MacArthur and Wilson 1967; Vellend 2016), its consequences for mediating the relative importance of selection and drift remain ungeneralizable (Catano et al. 2017; Lu 2021). For example, higher immigration was shown to decrease selection in marine communities (Loke and Chisholm 2023) but increase selection in terrestrial plant communities (Ron et al. 2018). Also, a meta‐analysis of experiments found higher seed arrival caused effects ranging from stronger selection (causing either homogenization or differentiation in plant communities) to patterns not different from what would be expected from randomly sampling species from the regional pool (Catano et al. 2017).

Such variable effects of immigration on assembly processes may depend on the number of species comprising the regional biota—regional species pool size (Conradi and Kollmann 2016). Larger species pools are often invoked to provide a regional explanation for higher local species diversity (Harrison and Cornell 2008), but alternative predictions for how species pool size alters ecological selection and drift remain unresolved. For example, immigration from larger species pools can enhance the role of selection by increasing the likelihood that species colonise communities at points along resource gradients where they are well adapted (Foster 2001; Germain et al. 2017; Questad and Foster 2008), thereby strengthening species–environment relationships (Figure 1A,Ei). Alternatively, immigration from larger species pools may increase drift; for example, when more arriving species increases the likelihood of historical contingencies, for example due to more possible arrival orders (Chase and Ryberg 2004; Vannette and Fukami 2017; Zou and Rudolf 2023), or the number of rare species that have smaller population sizes and thus more susceptible to chance events (Catano et al. 2021). Importantly, scenarios that increase drift are predicted to weaken species–environment relationships (Grman and Brudvig 2014) (Figure 1B,Eii).

These hypotheses and predictions remain unresolved because immigration and species pool size are confounded with each other and local conditions. More diverse regions have more rare species with small local population sizes (Soininen et al. 2012), making it challenging to disentangle regional pool effects from local ecological outcomes using correlative approaches (Harrison and Cornell 2008). While null models are widely used to determine how much local patterns deviate from those that could arise from randomly sampling a given species pool (Chase and Myers 2011; Kraft et al. 2011; Lessard et al. 2012), these approaches remain indirect, do not explicitly consider interactions with species pools, and may not reliably resolve selection versus drift (Tucker et al. 2016). To overcome limitations with correlative and indirect approaches, experiments are necessary that directly manipulate the factors thought to mediate selection and drift (Shoemaker et al. 2020), like dispersal (Loke and Chisholm 2023; Ron et al. 2018) or community size (Gilbert and Levine 2017). Such experiments can advance our understanding of fundamental processes, but thus far have been limited to relatively simple conditions, such as small‐scale mesocosms without environmental heterogeneity (Ron et al. 2018) or within a single region where species pool influences cannot be examined (Loke and Chisholm 2023). Therefore, it remains unknown the extent to which regional context mediates local ecological processes and the distribution of biodiversity at broad scales in natural ecosystems where environmental gradients are ubiquitous (Soininen 2014). Implementing controlled experiments across broader scales in the context of heterogeneous natural conditions is rare (Catano et al. 2021; Germain et al. 2017), but can reveal novel insights necessary to bridge theory with predictions of biodiversity change in natural ecosystems (Palmer et al. 1997).

We conducted a large‐scale experiment to quantify the extent to which regional contexts—those due to differences in the species pool size and immigration—mediate selection and drift structuring variation in plant diversity. Our experiment occurs in the context of grassland restoration, which allows our study to test theory with an unusual degree of realism (Temperton et al. 2004; Young et al. 2001). Regional pools and immigration are notoriously difficult to define (Lessard et al. 2012), but can be precisely quantified in restoration through the composition and density of seeds sown (Carter and Blair 2012; Grman and Brudvig 2014). We factorially manipulated species pool size (8 vs. 30 species) and immigration (270, 485, 700, 970 seeds m^−2^) by sowing seeds in 12 sites and 576 plots distributed across a landscape soil moisture gradient. We then tracked changes in the composition and relative abundance of the seeded species over 5 years. We evaluated three key predictions of the hypothesis that higher immigration and species pool size increase the importance of selection relative to drift (Figure 1); where drift is predicted to (1) cause divergence in species composition across communities (increase spatial *β*‐diversity over time); (2) reduce species diversity within communities (*α*‐diversity) and (3) reduce the amount of variation in species composition explained by the soil moisture gradient (weaken species‐sorting). Our study combines the advantages of controlled experiments with the reality of natural, complex ecosystems; therefore, offering unique opportunities to disentangle regional and local mechanisms that cause variation in plant species composition across scales, with implications for ecosystem restoration.

## 2 Materials and Methods

### 2.1 Experimental Community Assembly

We established 12 sites spanning a natural, landscape‐scale soil moisture gradient (xeric to mesic grasslands) in southern MI, USA (Michigan State University's Kellogg Biological Station—Lux Arbor Reserve and Fort Custer Training Center). Soil moisture is a key environmental gradient in grasslands that affects primary productivity, competitive interactions between plants, and plant species composition in natural and restored prairies (Catano et al. 2022; Houseman and Gross 2011; Knapp et al. 1998). To deplete the seed bank and prepare the soil for seed sowing, we applied two applications of broad‐spectrum herbicide followed by soil disturbance (tillage or hay‐raking depending on site conditions) at each site in 2017 (Packard and Mutel 2005). At each site, we established 48 5 × 5 m (25 m^2^) experimental plots. We quantified soil moisture in the first growing season by aggregating eight 20‐cm deep soil cores collected from the perimeter of each plot, then calculated soil water holding capacity as the proportionate difference between wet and oven‐dry weight (Grman et al. 2013) (Figure S1).

We manipulated immigration (4 levels: 270, 485, 700 and 970 seeds added per m^2^) crossed with species pool size (2 levels: 8, 30 species), with the eight treatments applied randomly to plots within each of the 12 sites (576 plots; 6 plots per treatment per site). This factorial, randomised design allowed us to quantify the extent to which treatment effects are robust to unmeasured, natural variation in site or landscape conditions. We applied treatments to each plot by hand‐sowing seeds of native prairie plants obtained locally (Native Connections; Three Rivers, MI, USA). To provide realism, the treatment levels were informed by two criteria: (1) models from past observational research on restored prairies that suggest levels of species pool size and immigration that are predicted to influence selection and drift (Grman et al. 2013; Grman and Brudvig 2014) and (2) in consultation with practitioners to span a range of seed sowing densities typically used in prairie restoration. Because variation in species identities and regional abundance distributions can create differences in community composition simply due to sampling effects (Vellend et al. 2014), we standardised seed mixes for each treatment, where species composition and relative abundance were fixed (equal number of individuals, i.e., pure live seed, for each species). The composition of the small species pool treatment is a subset of the large species pool treatment, stratified by proportions of major functional groups (Table S1), so that both species pool treatments encompass species typical of restored prairies in our region spanning xeric, mesic and wet‐mesic sites. This nested species pool design is important for three key reasons. First, the range of functional strategies in each pool provides opportunities for species to sort along the soil moisture gradient spanned by the 12 sites, regardless of the number of species. Second, the nested species pools ensure both pools include the regionally dominant competitors in this system (C_4_ bunchgrasses *Andropogon gerardii* and *Schizachyrium scoparium* ), where the larger pool adds more subordinate species that are typically rarer in tallgrass prairies. Third, this nested approach matches how restoration decisions are typically made, allowing our results to be more directly translatable to real‐world conservation strategies.

Due to the rarity of native prairies in the study region and resulting absence of prairie propagule sources, a strong link can be made between sown prairie seeds and the resulting community of prairie species (Grman and Brudvig 2014). To help sown species establish, all sites were mowed during the first two growing seasons after we finished sampling. Prescribed burns were conducted in the spring prior to the fifth growing season to mimic natural fire regimes that maintain native tallgrass prairies (Packard and Mutel 2005). To minimise edge effects and prevent species spill‐over between experimental plots, we created 1‐m alleys between plots and maintained a 10‐m buffer around the edge of each site.

### 2.2 Quantifying Plant Diversity Patterns

To measure plant community composition, we quantified the percent cover of all plant species in 1 × 1‐m quadrats at the centre of each 5 × 5‐m plot. We quantified plant species composition annually at peak biomass (July) over the first five growing seasons: 2018–2022 (*n* = 2880 plots). The 5‐year timeframe allows us to detect key mechanisms of selection that emerge during and after establishment (Tilman 2004). To provide a direct link between our experimental manipulations of species pool size and immigration, we focus analysis on the seeded species within each plot; however, we also consider relationships between treatments and nonseeded species to understand their potential influences.

We quantified local diversity of seeded species (*α*‐diversity) in each 1 × 1‐m plot in each year as the effective number of species using the exponential of Shannon entropy (Hill number *q* = 1) (Hill 1973). This diversity metric is ideal because it weights species according to their abundance and allows us to assess treatment effects on local diversity due to changes in richness and relative abundance (Jost 2006), which is important to assess effects of drift that increase extinction and/or dominance (i.e., more uneven relative abundances). We quantified spatial variation in seeded species composition (spatial *β*‐diversity) in each year by calculating Bray–Curtis dissimilarity among plots within each site for each treatment. We then partitioned Bray–Curtis dissimilarity into its unique components—balanced variation in abundance (βbal) and abundance gradients (βgra), which are abundance‐based analogues to turnover and nestedness (Baselga 2013). Treatment effects on βbal reflect changes caused by individuals being replaced by individuals of other species, whereas effects on βgra reflect changes in *β*‐diversity caused only by differences in the total number of individuals.

### 2.3 Data Analysis

We quantified variation in the distribution of plant diversity as the changes in *α*‐diversity and spatial *β*‐diversity over 5 years. Because raw dissimilarity values are not independent, we first quantified *β*‐diversity as multivariate dispersion (Anderson 2006)—the distance of each local community to the centroid (spatial median in principal coordinate space) of all communities in a treatment. Increasing dispersion over time within a treatment indicates species compositions among local communities are diverging, whereas decreasing dispersion indicates compositions of local communities are converging (Anderson et al. 2011). To test the hypothesis that species pool size and immigration interact to cause variation in community assembly, we fitted a linear mixed model using Restricted Maximum Likelihood and Satterthwaite degrees of freedom. *β*‐diversity and *α*‐diversity were fitted with a three‐way interaction between the fixed effects species pool size, immigration and year with random intercepts for site and a random slope for year. We standardised input variables (mean centred and divided by two standard deviations) in the models to put predictors on a common scale and to make main effects interpretable in the presence of interactions (Gelman 2008). Therefore, the effects are the responses of *β*‐diversity and *α*‐diversity to two standard deviations change in the predictor while all other predictors are at their means. We assessed model fits with a simulation‐based approach to calculate scaled residuals (Dunn and Smyth 1996; Gelman and Hill 2007). We square root transformed *β*‐diversity and log transformed *α*‐diversity to ensure scaled residuals were normally distributed and uniform across model predictions.

To test hypotheses for how immigration and species pool size alter selection relative to drift, we used distance‐based redundancy analysis (Legendre and Anderson 1999) to calculate the percentage of variation in composition (*β*‐diversity and its turnover vs. nestedness components) in each treatment explained by the soil moisture gradient. We then quantified treatment effects using linear models. Because the soil moisture gradient is the same across all treatments, higher variation explained by the soil gradient indicates treatments increased species sorting relative to treatments with lower variation explained, which indicates greater relative importance of drift. Importantly, soil moisture structures plant composition directly through environmental tolerance and indirectly by altering productivity and diversity of competitors; therefore, we take an integrative view of species sorting where the composition of seeded species is jointly influenced by both abiotic (soil moisture) and biotic (competition with nonseeded species) environments (Cadotte and Tucker 2017). All responses were log‐transformed to normalise residuals and ensure constant error variances. We conducted analyses in R version 4.2.2 (R Core Team 2022) using packages lme4 (Bates et al. 2015) to fit linear mixed effects models, DHARMa v0.4.6 (Hartig 2022) to run simulations to assess model fits, betapart v1.6 (Baselga and Orme 2012) to calculate *β*‐diversity partitions and vegan v2.6–4 (Oksanen 2022) to quantify multivariate dispersion and perform distance‐based redundancy analyses.

## 3 Results

We found that immigration (number of seeds sown) and regional species pool size (number of species sown) jointly altered the local community assembly processes that give rise to variation in plant diversity. First, higher immigration increased initial community size (Figure S2) and decreased spatial *β*‐diversity in seeded species across local communities (std. effect = −0.03, 95% CI [−0.05, −0.02], Figure 2B), aligned with our predictions in Figure 1C. In contrast, we found that larger species pools increased spatial *β*‐diversity (std. effect = 0.06 [0.05, 0.07], Figure 2C). Surprisingly, species composition converged across plots within each treatment (std. effect = −0.08 [−0.11, −0.05], Figures 2A,D, S3 and Table S2), inconsistent with predictions for drift that would cause composition to diverge.

**FIGURE 3:**
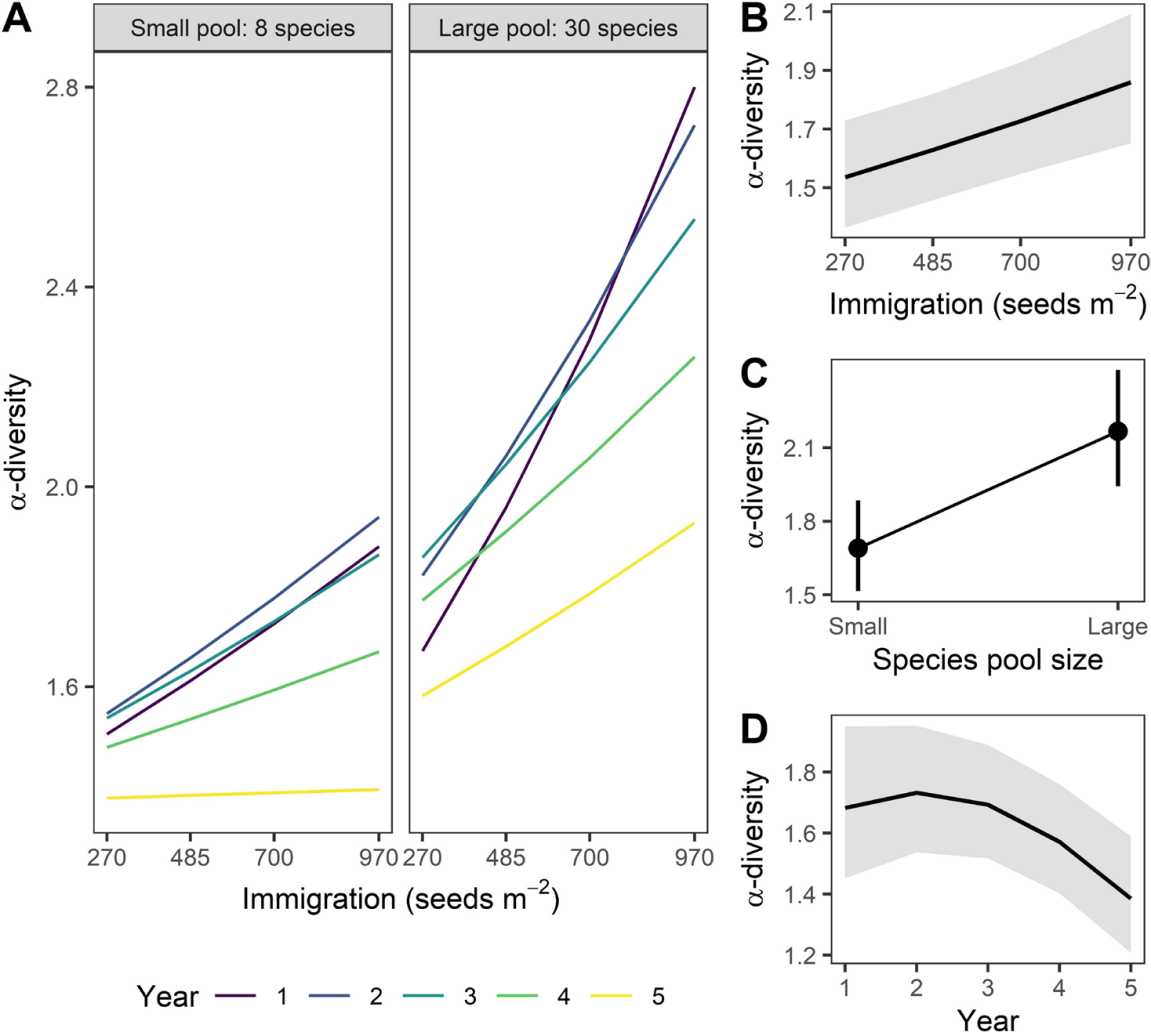
Effects of Immigration, Species pool size and Time (Year) on local (*α*) diversity (effective number of species; Shannon Diversity). The effect of Immigration varies with Species pool size and over time (Year) (A). Immigration (B), Species pool size (C) and Year (D) are the model predictions (marginal effects) conditioned on the other input variables fixed at their mean, with 95% CIs. All main effects are statistically significant (Table S2). The model was fitted to log transformed *α*‐diversity, with random slopes for years (*n* = 5) and random intercepts for sites (*n* = 12), and results plotted on the scale of the original untransformed data (*n* = 2606). Model predictions and data for each site are shown in Figure S4.

Second, higher immigration increased seeded species *α*‐diversity within communities (std. effect = 0.11 [0.07, 0.16], Figure 3B), aligned with predictions in Figure 1D. Immigration increased *α*‐diversity more when seeds were sown from larger species pools (Immigration × Species pool: std. effect = 0.13 [0.07, 0.19]). However, the importance of immigration on local *α*‐diversity declined over time (Immigration × Year: std. effect = −2.82 [−5.01, −0.63]) (Figures 3A, S4 and Table S3) suggesting that community assembly is more structured by dispersal limitation early in succession. Independent of immigration, *α*‐diversity increased with species pool size (std. effect = 0.25 [0.21, 0.28], Figure 3C) and declined over time (std. effect = −3.6 [−6.8, −0.03], Figure 3D). Importantly, species pool effects were statistically clear for both spatial *β*‐diversity (*t* = −2.30, df = 2566, *p* = 0.02) and *α*‐diversity (*t* = −11.12, df = 2556, *p* < 0.001) in the treatment contrasts with similar seed arrival per species and community size (large pool/high immigration × small pool/low immigration) (Figure S2 and Table S6). This result suggests that greater *α*‐ and spatial *β*‐diversity due to larger species pools was due to a combination of lower mean colonisation per species and differences in regional diversity.

**FIGURE 4:**
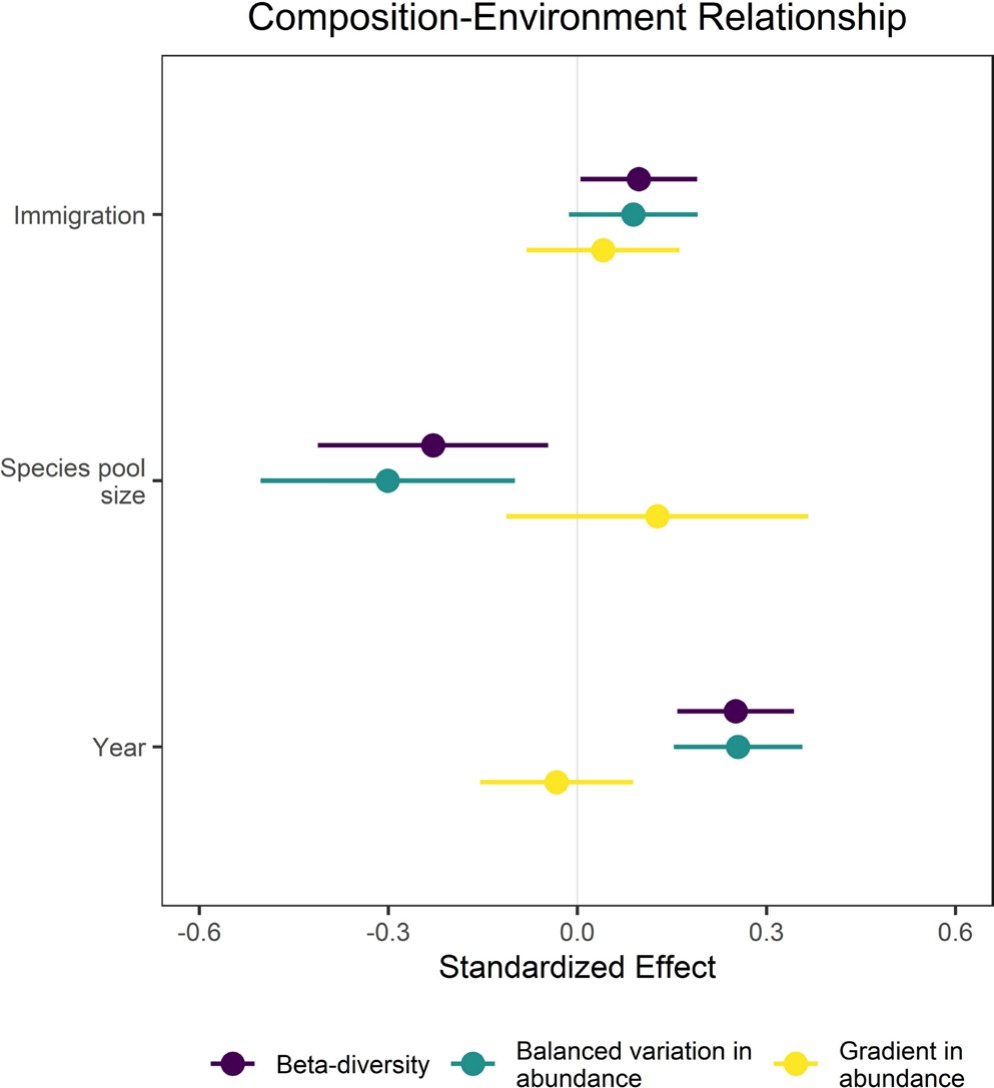
Effects of Immigration, Species pool size and Time (Year) on the strength of species sorting across a soil moisture gradient. Points are standardised model coefficients (mean‐centred and divided by two standard deviations) with 95% CIs. Negative values indicate the treatment weakened the importance of the soil moisture gradient in explaining dissimilarity in species composition. We fitted models to total variation (*β*‐diversity) and its components due to balanced variation in abundance (akin to turnover in species composition and relative abundance) versus those caused by gradients in total abundance (*n* = 40 for each *β*‐diversity response variable).

Finally, regional context altered the relationship between species composition and the landscape soil moisture gradient. We found that 54% of the variation in composition–environment relationships was explained by species pool size, immigration and time (*F*_3,36_ = 13.86, *p* < 0.001, *R* ^2^ = 0.54; Figure 4). The amount of compositional variation explained by the soil moisture gradient decreased with species pool size (std. effect = −0.29 [−0.56, −0.02]), increased with immigration (std. effect = 0.31 [0.04, 0.59]) and increased over time (std. effect = 0.76 [0.48, 1.03]). Consistent with the species sorting mechanism, changes in the strength of composition–environment relationships were primarily caused by differences in species turnover and relative abundances (βbal: *F*_3,36_ = 13.67, *p* < 0.001, *R* ^2^ = 0.53), not the total number of individuals (βgra: *F*_3,36_ = 0.80, *p* < 0.500, *R* ^2^ = 0.06) (Figure 4 and Table S4).

## 4 Discussion

The importance of regional processes for community assembly and the distribution of biodiversity has been debated for decades (Harmon and Harrison 2015; Harrison and Cornell 2008; Rabosky and Hurlbert 2015; Ricklefs 2008), yet experimental evidence from large‐scale, natural ecosystems is rare (Germain et al. 2017). By conducting a landscape‐scale experiment in restored grasslands, we found that greater immigration and smaller species pools shifted plant community assembly processes towards greater selection relative to drift. This shift occurred due to strengthening species sorting along the landscape soil moisture gradient, but also reduced plant *α*‐diversity and spatial *β*‐diversity over time. Our results suggest that regional constraints on colonisation make assembly more variable, but help maintain diversity by limiting biotic homogenisation and allowing the persistence of more rare species in the region.

Our results support the hypothesis that species pool size and immigration alter the importance of two interrelated selection mechanisms—local dominance and landscape‐scale species sorting. First, higher immigration increased *α*‐diversity (Figure 3B) and reduced *β*‐diversity (Figure 2B) as more species established in communities, consistent with other studies showing communities are primarily dispersal limited following disturbance (Brown and Fridley 2003; Catano et al. 2017; Myers and Harms 2009). However, *α*‐diversity declined over time as communities converged towards similar states (Figures 2D and 3D) dominated by the strongest competitor in the pool—Big bluestem (*Adropogon gerardii*) (Figures S5 and S6). Big bluestem is a native bunchgrass and strong competitor for nitrogen and light (Dybzinski and Tilman 2007), reaching heights greater than 2 m while expanding clonally to monopolise space, which can lead to competitive exclusion (Grman et al. 2020) and biotic homogenisation in grasslands (Catano et al. 2022). Immigration from the larger species pool reduced big bluestem dominance, which caused greater local seeded species diversity, and limited both diversity loss (Figure 3A) and species replacement over time (Figure S7). Therefore, colonisation history and initial conditions can alter assembly trajectories (Cadotte 2023). These results suggest that communities assembled from the large species pool remain dispersal assembled (positive immigration—*α*‐diversity relationship) over all 5 years following colonisation, whereas communities assembled from the small species pool became more structured by selection over time (flat immigration—*α*‐diversity relationship) (Loke and Chisholm 2023). To date, the role of dispersal in balancing selection versus drift has remained ungeneralisable (Catano et al. 2017; Lu 2021). Our results provide evidence that variable effects of dispersal on community assembly processes may be reconciled by considering the modifying effects of regional species pools.

Second, immigration and species pool size altered the role of species sorting at the landscape scale. We found that smaller species pools and higher immigration increased the importance of soil moisture for structuring the spatial variation in composition (spatial *β*‐diversity). This resulted from more turnover in species composition (*β*bal), not simply sampling effects due to differences in the number of individuals (*β*gra) (Figure 4). These results support the effective community size hypothesis (Ron et al. 2018), which predicts that more individuals will increase the strength of competition mediated by species' fitness differences (Orrock and Fletcher Jr. 2005; Orrock and Watling 2010). While prairie species are known to exhibit differential fitness across soil resource gradients according to physiological constraints and environmental tolerance (Catano et al. 2022; Zirbel et al. 2017), soil moisture also increased the cover of nonseeded species that colonised from the seedbank and surrounding area (Figure S8). Therefore, we consider stronger selection due to species sorting in our study to be imposed by the joint influence of the abiotic (soil moisture) and biotic (competition with nonseeded species) environment (Cadotte and Tucker 2017). These results provide evidence that species pool size and immigration can mediate the strength of selection by increasing the effective community size across environmental gradients in natural ecosystems (Catano et al. 2022; Zirbel et al. 2017).

In contrast, lower immigration and larger species pools eroded the importance of soil moisture for structuring plant diversity patterns. Larger species pools and lower immigration could increase the importance of at least two nonmutually exclusive mechanisms: chance colonisation and drift due to bottlenecks in initial community size. Consistent with chance colonisation, reducing immigration and increasing species pool size caused greater plant *β*‐diversity (Figure 2). Lower colonisation of big bluestem reduced its establishment success (Figure S9) and limited its increase in cover over time (Figure S6), thus allowing weaker competitors to establish and increase in cover (Figure S10). This result is consistent with the hypothesis that lower immigration and larger species pools mitigate local competitive exclusion (Hurtt and Pacala 1995), thereby allowing more subordinate species to establish and increase variation in species composition, which persisted over the first 5 years. These results suggest a trade‐off predicted by spatial coexistence models (Shoemaker and Melbourne 2016), where regional constraints on colonisation make assembly more stochastic while also helping to maintain diversity at the regional scale by limiting biotic homogenisation.

Despite lower immigration and larger species pools causing lower community size (Figure S2), surprisingly we did not find evidence that drift subsequently became more important. Following the initiation of the experiment, compositions converged over the next 5 years within all treatments (Figure 2), inconsistent with models (Orrock and Fletcher Jr. 2005; Orrock and Watling 2010), experiments (Gilbert and Levine 2017; Ron et al. 2018) and correlative studies (Jacobi and Siqueira 2023; Siqueira et al. 2020) that predict lower community size will increase compositional divergence through drift. Moreover, the soil moisture gradient became more important in explaining variation in species composition over the 5 years of our experiment in all species pool and immigration treatments (Figure 4). Though drift is increasingly realised to be an important process in theory (Vellend 2016) and empirical studies (Gilbert and Levine 2017), its role in naturally complex ecosystems remains rarely quantified. In our experiment, compositional convergence and the strengthening of the composition–environment relationship suggest that though drift appeared more important early in succession, selection eventually overrode drift even at low community sizes. We expect these patterns to persist or strengthen with time due to priority effects gained by dominant species early in assembly (Fukami 2015), such as niche construction by big bluestem and resulting biomass‐fire severity feedbacks (Catano et al. 2023), and the long‐lived nature of perennial grassland plants (Cadotte 2023).

An important advance of our study is that our experimental design allowed us to disentangle the effects of regional species pool size from those due to differences in species abundance distributions and the total number of individuals across sites with different local environmental conditions—key features of diversity that are inseparable in observational studies (Engel et al. 2021; McGill 2011). As a result, we found that the effects of species pool size on *β*‐diversity remained significant in contrasts where average seed arrival per species and local community size were controlled experimentally, which suggests species pool effects operate beyond constraints on local populations. However, larger species pools did not increase species sorting across the landscape soil moisture gradient (Figure 4). Instead, species pool effects in our study could arise through nonspatial mechanisms. For example, larger species pools tend to have more species that overlap in ecological strategies, which can make assembly sensitive to historical contingencies and/or make outcomes of competition more unpredictable (Chase 2003; Spasojevic et al. 2018). Also, larger species pools and lower immigration permitted more nonseeded species to achieve relative covers exceeding 60% on average (Figure S8), potentially contributing to variation through unmeasured interactions between seeded and invading species. While it is impossible to quantify all the potential species pool and local mechanisms in this study, we provide some of the first large‐scale experimental evidence that regional contexts mediated the balance of selection and drift underpinning patterns of plant diversity across scales.

We expect the results of this study to be generalisable across many natural and managed ecosystems that are sensitive to initial immigration events; for example, ecosystem restoration (Palmer et al. 1997) or colonisation and primary succession following disturbance (Connell 1978). Future studies could expand upon this work by integrating more complex dispersal and species pool scenarios typical of some other systems. Studies should go beyond taxonomic dimensions of the pool; for example, manipulating functional attributes of the pool could help reveal species pool effects that emerge due to differences in evolutionary or biogeographic history, regardless of species pool size (Spasojevic et al. 2018). Additionally, considering dispersal traits in the context of species pools could help advance our understanding of competition–colonisation trade‐offs in community assembly. Moreover, future studies would benefit from considering more complex dispersal scenarios, such as differences in the directionality or frequency of dispersal in patchy metacommunities (Grainger and Gilbert 2016) or immigration in the context of biological invasions into communities already saturated with species. Finally, while our study manipulated species pool size through nestedness, studies that randomise the species in seed mixes could further disentangle the role of species pool richness from differences in composition.

By conducting our experiment in the context of restored prairie communities, our findings are especially informative for ecosystem restoration—a key strategy that is the centrepiece of international initiatives to reverse ecosystem degradation and biodiversity loss (Suding et al. 2015). Restoration outcomes are notoriously variable (Atkinson et al. 2022) and resolving the drivers of this variability is a pressing research need (Brudvig et al. 2017). Our study shows that variability among local restoration outcomes results from three primary factors: environmental heterogeneity, diversity and density of the seed mixes used to initiate restoration, and their influence on succession. By understanding how these factors mediate the predictability of restoration outcomes, this information can be integrated into models needed to guide restoration actions and maintain plant diversity (Brudvig and Catano 2024; Cadotte 2023). For example, when determining optimal seed mix design to balance competing goals (Barak et al. 2022), our results suggest that higher seed mix richness would increase local diversity, but also variability among restorations. Higher seeding density could increase the consistency among restorations, but at the cost of higher competitive dominance that suppresses diversity locally and regionally (Grman et al. 2020). Thus, these processes affect the predictability of outcomes and can have emergent effects on the functions and services of restored ecosystems (Catano et al. 2023).

## 5 Conclusion

Understanding the interplay between regional and local processes has long been a primary challenge at the intersection of ecological theory and applications to solve the global biodiversity crisis. We show that ecological contingencies that make biodiversity change seem unpredictable can be understood more generally through broad‐scale, integrative approaches that explicitly recognise the interactions between regional and local processes. Our landscape‐scale experiment suggests the consequences of dispersal on the tension between ecological selection and drift may be a general phenomenon governing the assembly and distribution of plant diversity in natural ecosystems. Anthropogenic impacts are changing the structure of regional biotas through species invasions and homogenising effects of global changes (Mckinney and Lockwood 1999), which can modify how local communities respond to their environment (Olden et al. 2004; Spasojevic et al. 2018). By considering interactions of processes operating across regional and local scales, we can advance a unified theory of ecological communities and confront the complexities of biodiversity change head on to meet global conservation initiatives.

## Author Contributions

C.P.C. and L.A.B. designed the study; C.P.C. analysed data and wrote the first draft of the paper and all authors performed the research and contributed to revisions.

### Peer Review

The peer review history for this article is available at https://www.webofscience.com/api/gateway/wos/peer‐review/10.1111/ele.70095.

## Supporting information


Data S1.

## Data Availability

All data and code necessary to reproduce the results and figures in this paper are freely available: DOI: https://doi.org/10.5281/zenodo.11068682.
