# Supplementary material for: Regional Processes Mediate Ecological Selection and the Distribution of Plant Diversity Across Scales
Source: Ecol Lett. 2025 Mar 5;28(3):e70095. doi: 10.1111/ele.70095 (PMC11881218; doi:10.1111/ele.70095)
Supplement: Supplementary file 1 — Data S1. [file ELE-28-0-s001.pdf]

ECOLOGY LETTERS

Supporting Information for

Experimental evidence that regional processes mediate ecological selection and the distribution of plant diversity

Christopher P. Catano, Jonathan Bauer, Tyler Bassett, Eric Behrens, Lars A. Brudvig

Corresponding author: Christopher P. Catano,
Email: chcatano@gmail.com

This PDF file includes:

Figures S1 to S10
Tables S1 to S6

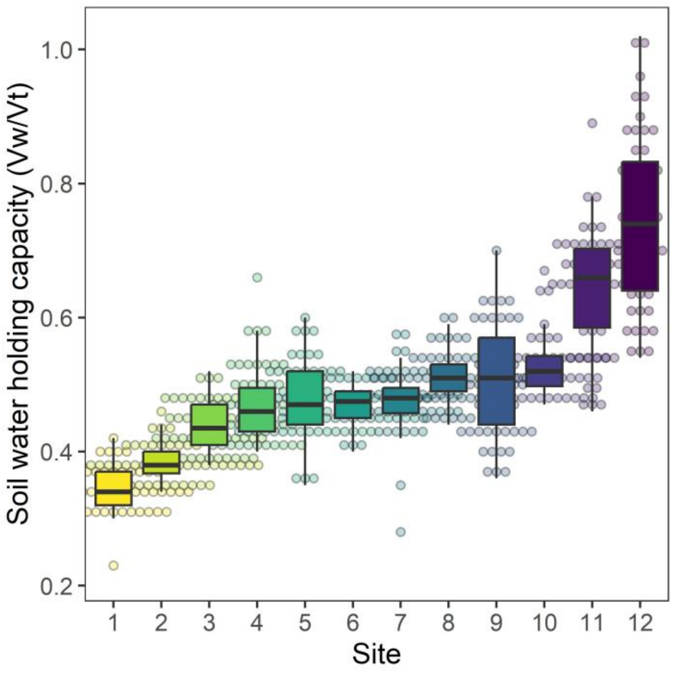

Fig. S1. Distribution of experimental sites across landscape-scale gradient in soil water holding capacity (volume of water / total volume of saturated soil). Soil water holding capacity is a key environmental gradient structuring turnover in plant species composition in tallgrass prairie ecosystems and is correlated with soil texture, organic matter, and soil nutrient availability.

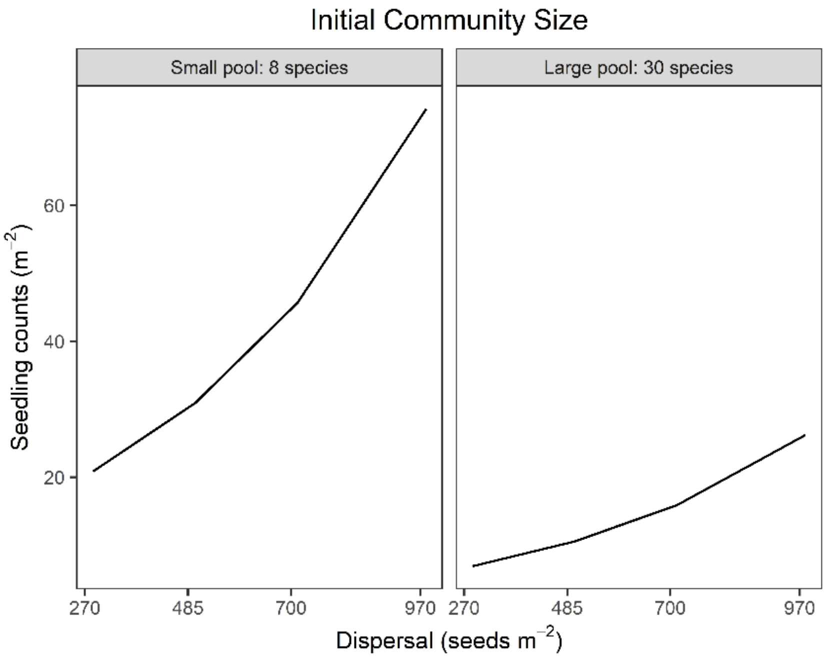

Fig. S2. Effects of species pool size and immigration treatments on initial community size (seedling counts of colonized species in the first year). Immigration (seed density) was standardized, and seedling counts were natural-log transformed prior to fitting models as described for diversity responses. See Table S6 for model coefficients.

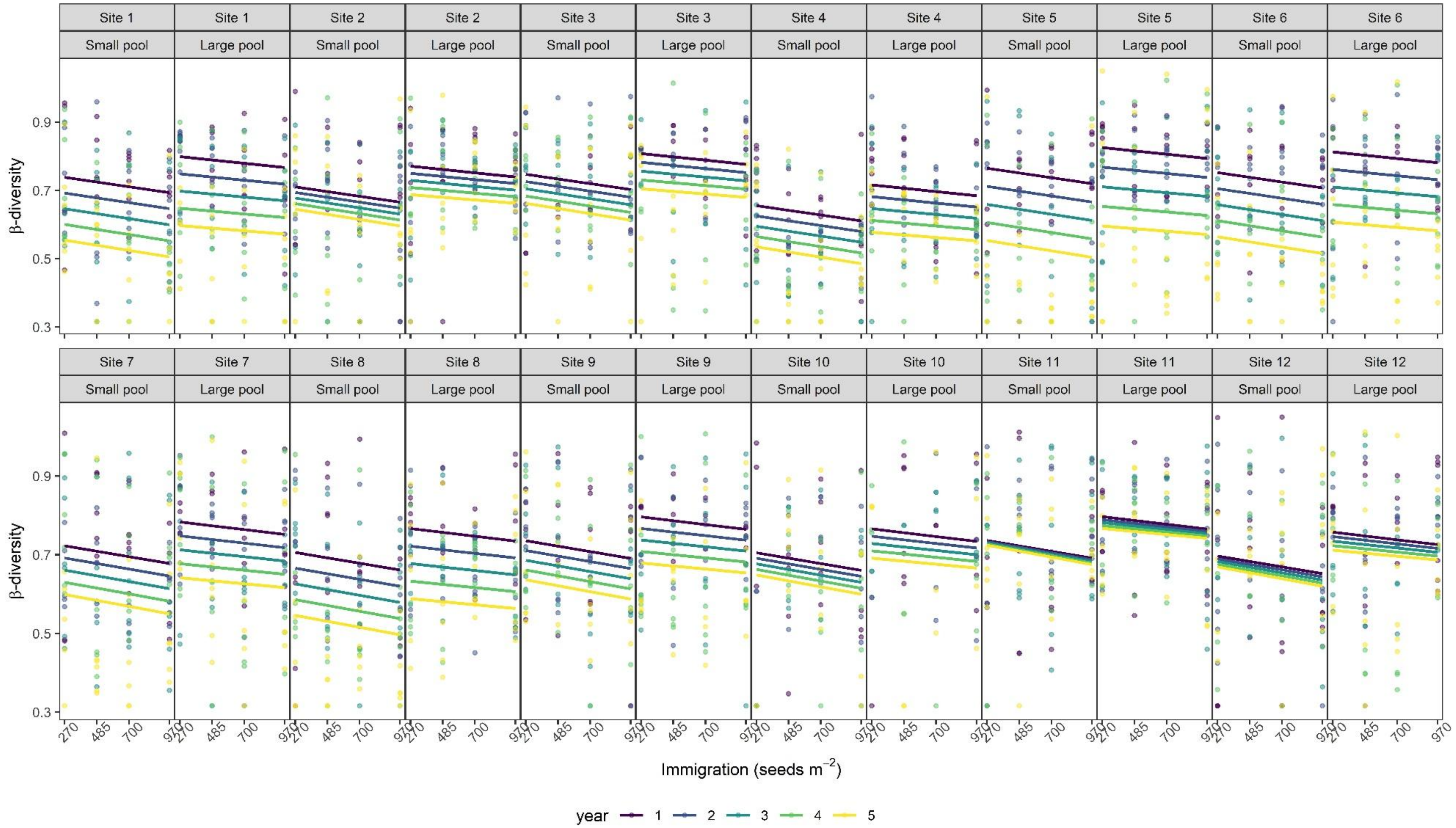

Fig. S3. Effects of Immigration, Species pool size, and Time (Year) on variation in plant community composition (β-diversity). Linear Mixed Effects model predictions are shown with the raw data for each level of the random effect (sites, n = 12). The model was fitted to square root transformed β-diversity and results are plotted on the scale of the original untransformed data (n = 2606).

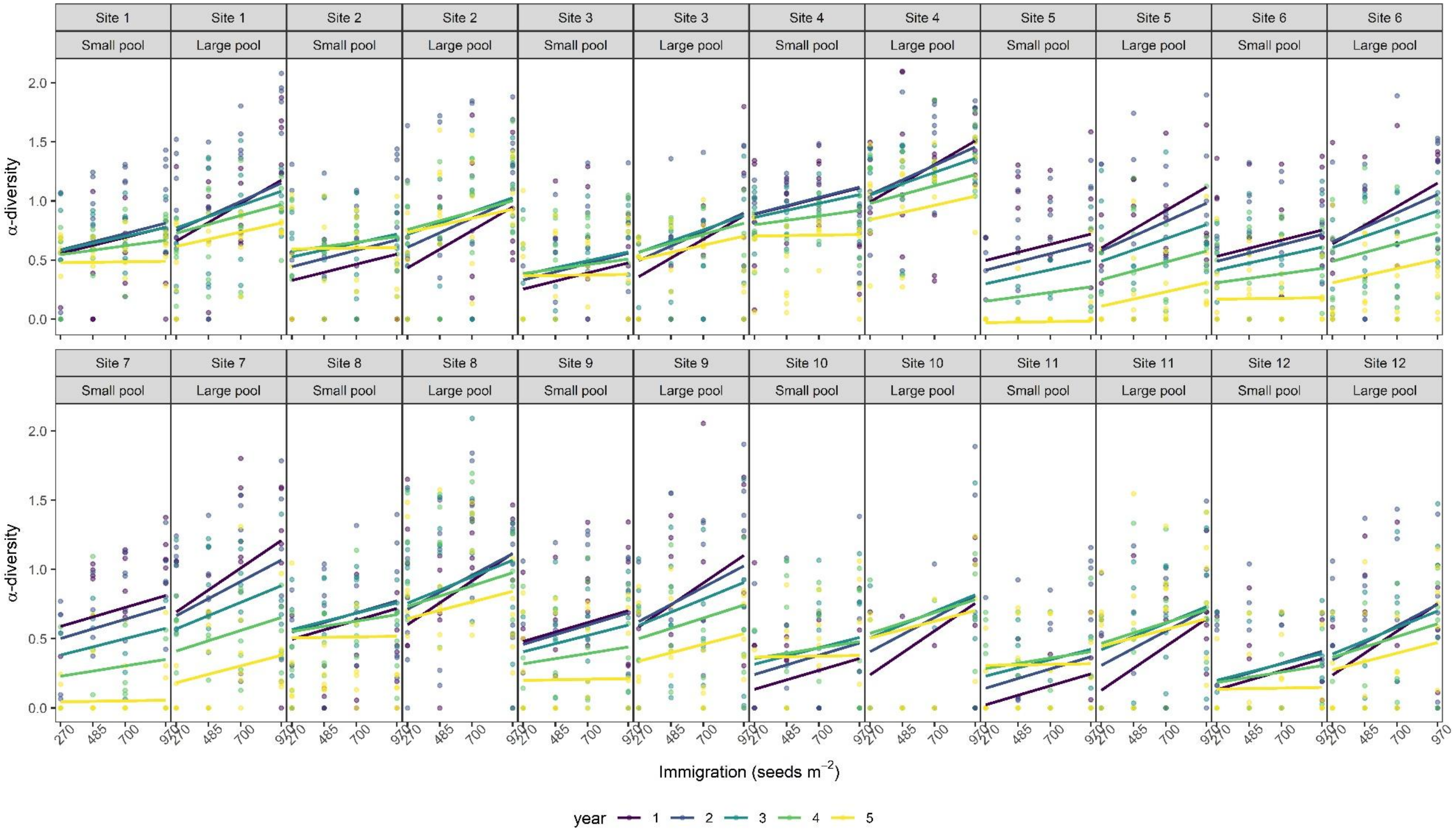

Fig. S4. Effects of Immigration, Species pool size, and Time (Year) on local (α) diversity (effective number of species; Shannon Diversity). Linear Mixed Effects model predictions are shown with the raw data for each level of the random effect (sites, *n* = 12). The model was fitted to log transformed α-diversity and results plotted on the scale of the original untransformed data (*n* = 2606).

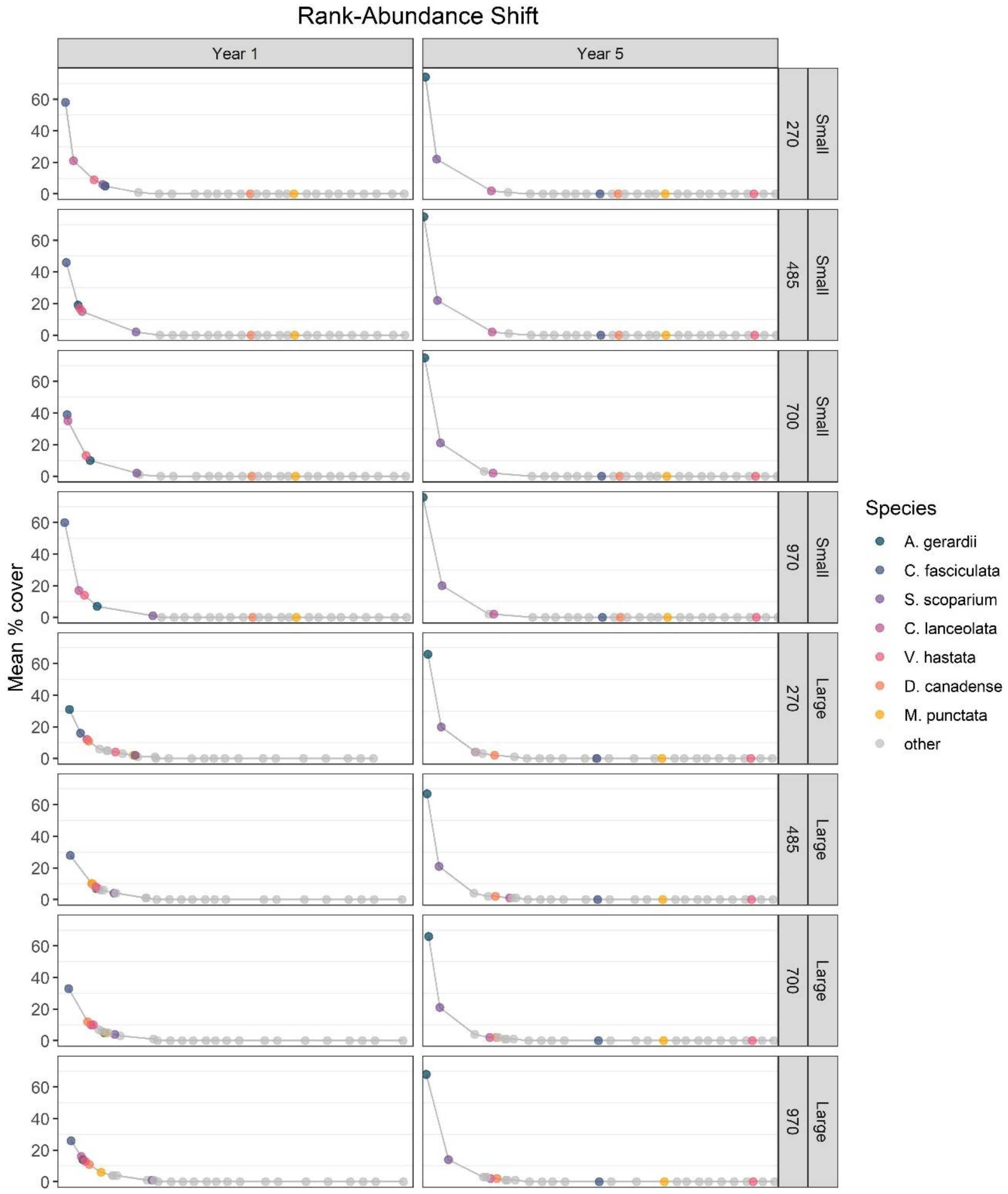

Fig. S5. Rank-abundance curves in first (Year 1) and last (Year 5) of the experiment for each treatment (Species pool size: Small vs Large; Immigration: 270 – 970 seeds/m^2). Percent covers are the means across all plots within a treatment. Only Species with % covers of at least 10% were shown. All other species are colored gray.

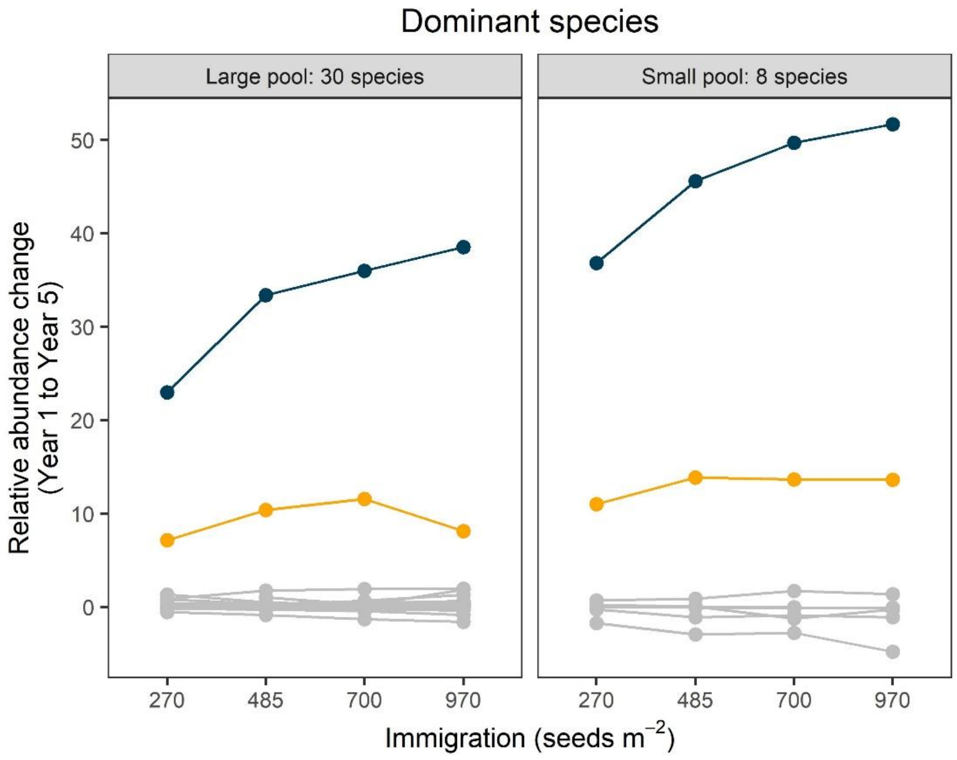

Fig. S6. Observed changes in relative abundance (cover) of seeded species (blue: *A. gerardii*; Yellow: *S. scoparium*; Grey: other seeded species) between the first and fifth year of the experiment. Seeded species are those included in the species pool and immigration treatments. Relative abundance was calculated at the treatment level (abundances were summed across all plots and sites within a treatment).

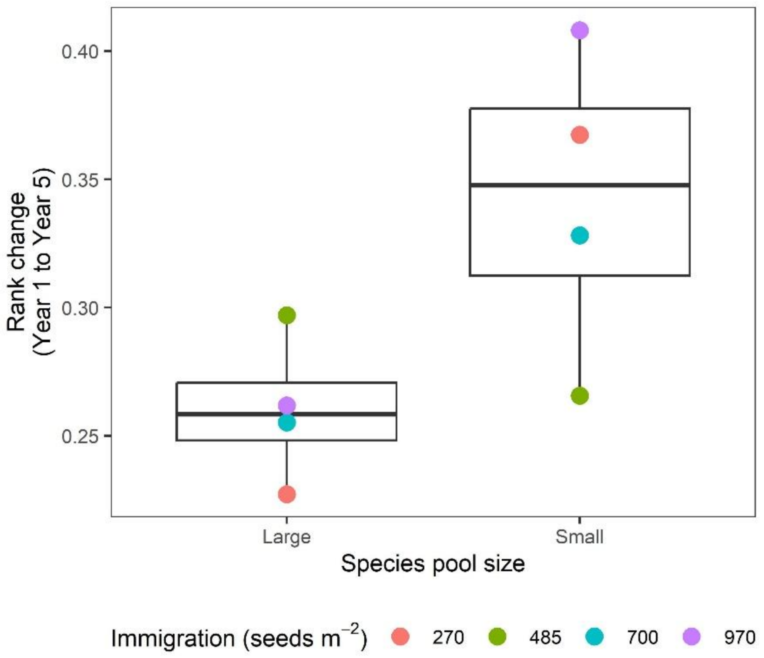

Fig. S7. Rank change (species reordering) within each Species pool by Immigration treatment. Relative abundance was calculated at the treatment level (covers were summed across all plots and sites within a treatment). Higher values indicate greater change in species abundance relative to each other, resulting in greater reordering of species ranks in the community, where the maximum possible rank change is 0.5.

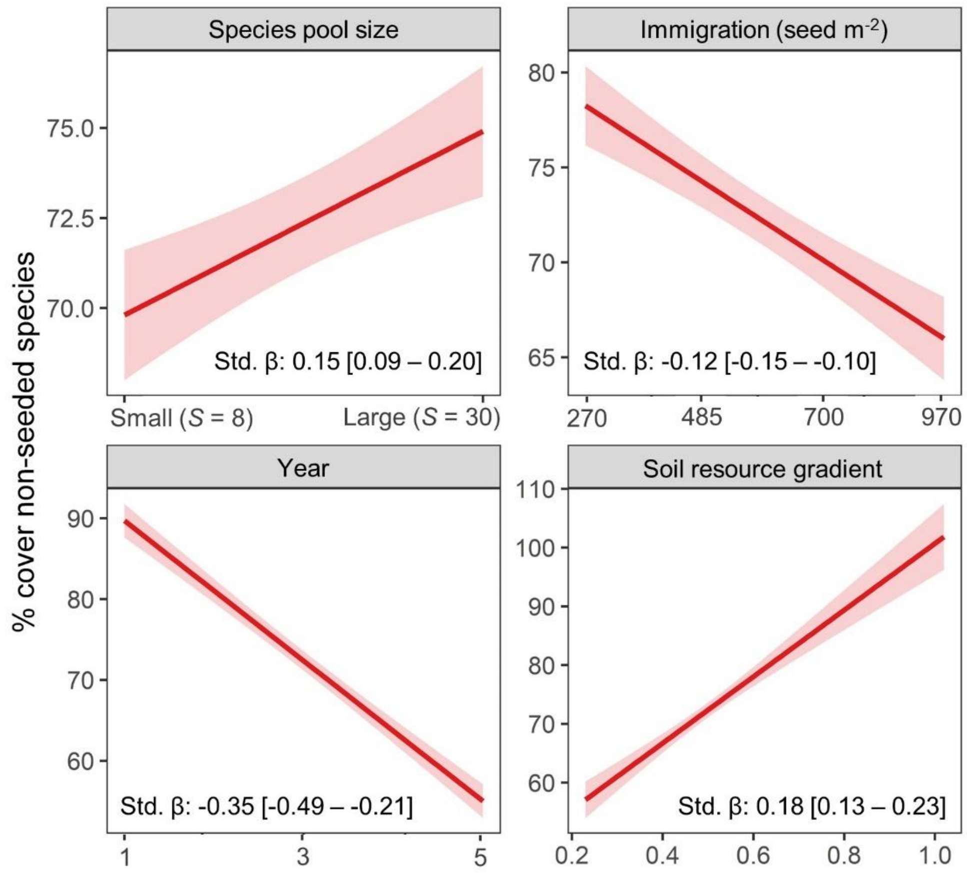

Fig. S8. Differences in cover of non-seeded species (those not in seed mixes that colonized naturally) in each plot. Lines are slopes of model coefficients and standard errors (standardized coefficients and their 95% confidence intervals are reported in each panel) from an additive linear mixed effects model with random intercept for site and random slope for year. Year and Immigration were standardized before fitting model.

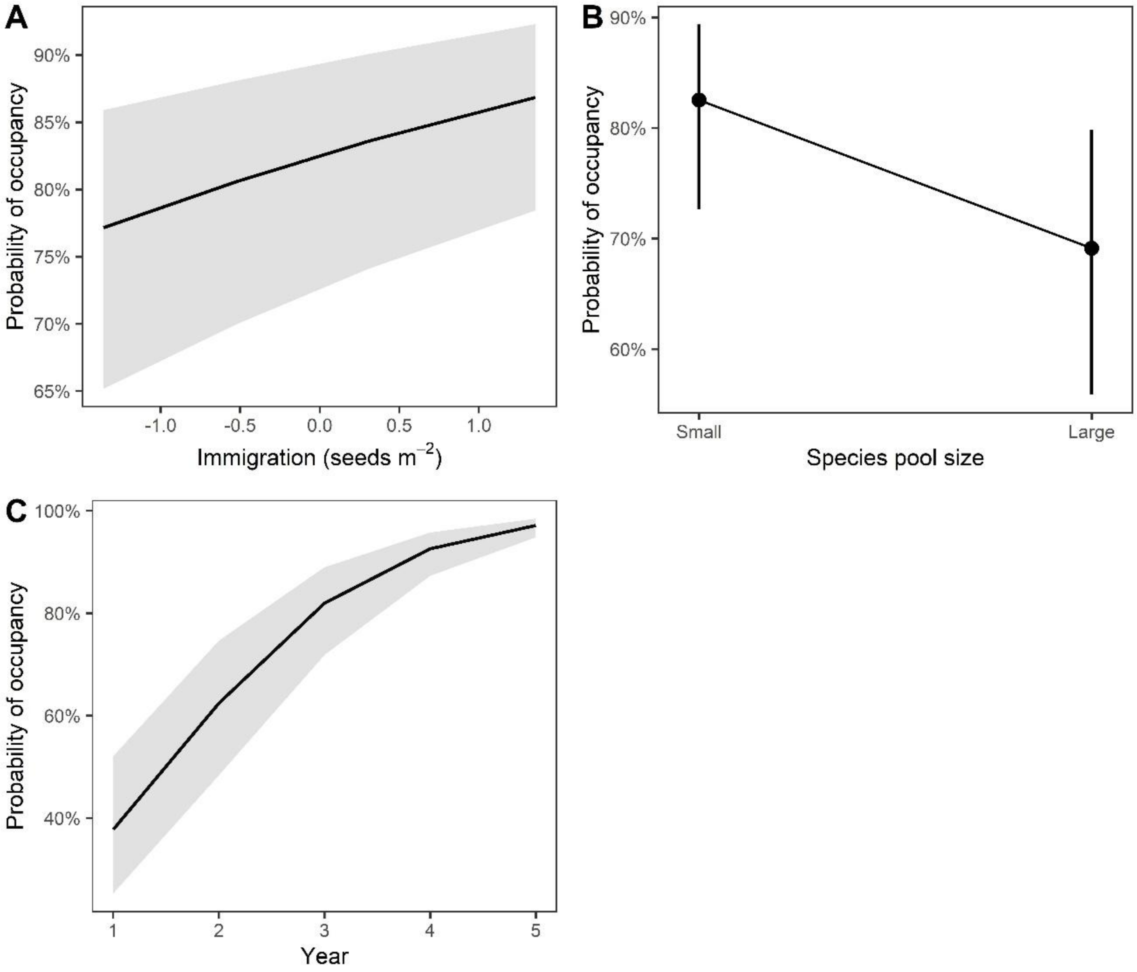

Fig. S9. Treatment and year effects on probability *A. gerardii* was detected in plots. Logistic regression model was fit to the data and each effect was statistically significant ($P < 0.001$). Plots are estimated marginal means while controlling for other variables.

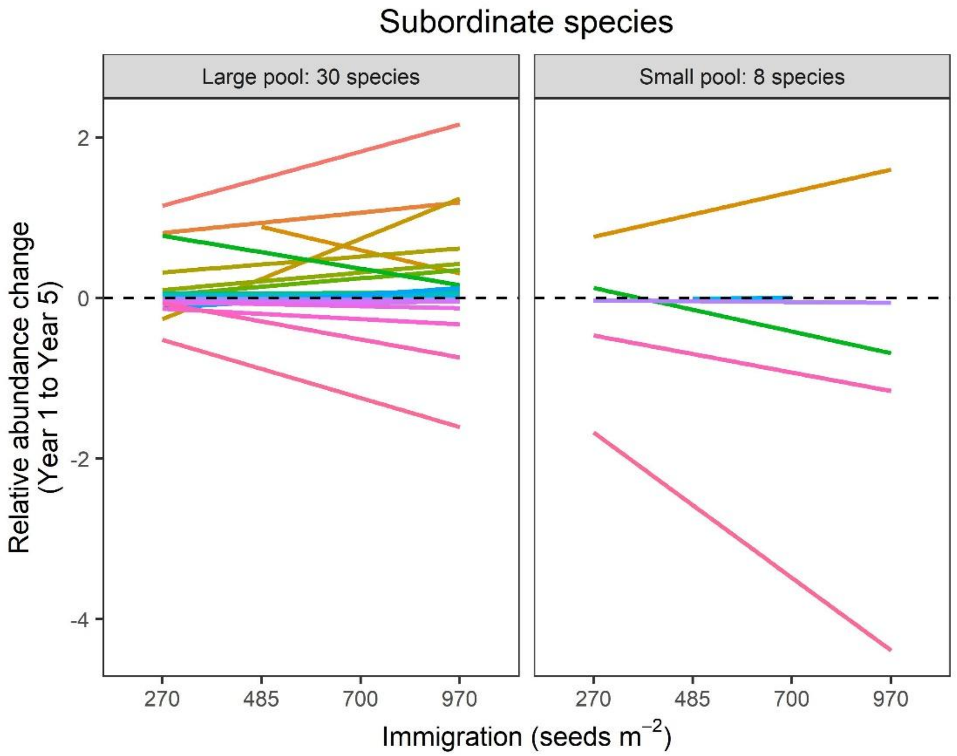

Fig. S10. Observed changes in relative abundance (cover) of subordinate seeded species (all but *A. gerardii* and *S. scoparium*) between the first and fifth year of the experiment, where colors correspond to different species (28 and 6 subordinate species in the large and small pools, respectively). Seeded species are those included in the species pool and immigration treatments. Relative abundance was calculated at the treatment level (covers were summed across all plots and sites within a treatment).

Table S1. Composition of the species pool treatments. All species are native to midwestern tallgrass prairie ecosystems and are representative of those typically considered for seed-based restoration. See footnote for descriptions of wetland indicator status.

Small species pool	Large species pool	Family	Functional group	Wetland Indicator Status
Coreopsis lanceolata	*Coreopsis lanceolata*	Asteraceae	Forb	FACU
Verbena hastata	*Verbena hastata*	Verbenaceae	Forb	FACW
Carex vulpinoidea	*Carex vulpinoidea*	Cyperaceae	Graminoid, sedge	FACW
Koeleria macrantha	*Koeleria macrantha*	Poaceae	Graminoid, C3	UPL
Andropogon gerardii	*Andropogon gerardii*	Poaceae	Graminoid, C4	FAC
Schizachyrium scoparium	*Schizachyrium scoparium*	Poaceae	Graminoid, C4	FACU
Astragalus canadensis	*Astragalus canadensis*	Fabaceae	Legume	FAC
Chamaecrista fasciculata	*Chamaecrista fasciculata*	Fabaceae	Legume	FACU
	Anemone canadensis	Ranunculaceae	Forb	FACW
	Anemone cylindrica	Ranunculaceae	Forb	UPL
	Symphyotrichum novae-angliae	Asteraceae	Forb	FACW
	Eryngium yuccifolium	Apiaceae	Forb	FAC
	Helenium autumnale	Asteraceae	Forb	FACW
	Brickellia eupatorioides	Asteraceae	Forb	UPL
	Monarda punctata	Lamiaceae	Forb	UPL
	Potentilla arguta	Rosaceae	Forb	None
	Drymocallis arguta	Rosaceae	Forb	FACU
	Pycnanthemum virginianum	Lamiaceae	Forb	FACW
	Rudbeckia hirta	Asteraceae	Forb	FACU
	Solidago rigida	Asteraceae	Forb	FACU
	Tradescantia ohiensis	Commelinaceae	Forb	FACU
	Verbena stricta	Verbenaceae	Forb	UPL
	Veronicastrum virginicum	Plantaginaceae	Forb	FAC
	Zizia aurea	Apiaceae	Forb	FAC
	Carex bicknellii	Cyperaceae	Graminoid, sedge	FACU
	Elymus canadensis	Poaceae	Graminoid, C3	FACU
	Spartina pectinata	Poaceae	Graminoid, C4	FACW
	Sporobolus cryptandrus	Poaceae	Graminoid, C4	FACU
	Baptisia alba	Fabaceae	Legume	FACU
	Dalea purpurea	Fabaceae	Legume	UPL
	Desmodium canadense	Fabaceae	Legume	FACU
	Lupinus perennis	Fabaceae	Legume	UPL
	Senna hebecarpa	Fabaceae	Legume	FACW

Indicator categories

**Indicator Code**	**Indicator Status**	**Designation**	**Comment**
OBL	Obligate Wetland	Hydrophyte	Almost always occur in wetlands
FACW	Facultative Wetland	Hydrophyte	Usually occur in wetlands, but may occur in non-wetlands
FAC	Facultative	Hydrophyte	Occur in wetlands and non-wetlands
FACU	Facultative Upland	Nonhydrophyte	Usually occur in non-wetlands, but may occur in wetlands
UPL	Obligate Upland	Nonhydrophyte	Almost never occur in wetlands

Citations:
USDA, NRCS. 2023. The PLANTS Database (http://plants.usda.gov, 11/02/2023). National Plant Data Team, Greensboro, NC USA.
U.S. Army Corps of Engineers 2020. National Wetland Plant List, version 3.5. http://wetland-plants.usace.army.mil/ U.S. Army Corps of Engineers Engineer Research and Development Center Cold Regions Research and Engineering Laboratory, Hanover, NH
Lichvar, R.W., N.C. Melvin, M.L. Butterwick, and W.N. Kirchner. 2012. National Wetland Plant List indicator rating definitions. U.S. Army Corps of Engineers, Engineer Research and Development Center, Cold Regions Research and Engineering Laboratory ERDC/CRREL TR-12-1.
Long Version of Indicator Definitions: https://wetland-plants.usace.army.mil/nwpl_static/data/DOC/NWPL/pubs/2012b_Lichvar_et_al.pdf

Table S2. Spatial β-diversity linear mixed model results fit using Restricted Maximum Likelihood and Satterthwaite degrees of freedom. β-diversity was square root transformed to linearize relationships and satisfy model assumptions. Input variables Immigration and Year were standardized by centering and dividing by two standard deviations prior to fitting model.

Predictors	*Estimates*	*std. Error*	*CI*	*Statistic*	*p*	*df*
(Intercept)	0.64	0.01	0.62 – 0.66	56.06	<0.001	2590
Species pool size	0.06	0.01	0.05 – 0.07	10.32	<0.001	2590
Immigration	-0.03	0.01	-0.05 – -0.02	-4.14	<0.001	2590
Year	-0.08	0.02	-0.11 – -0.05	-4.84	<0.001	2590
Species pool size × Immigration	0.01	0.01	-0.01 – 0.04	1.17	0.244	2590
Species pool size × Year	-0.01	0.01	-0.03 – 0.01	-0.75	0.452	2590
Immigration × Year	0.00	0.02	-0.04 – 0.03	-0.14	0.891	2590
(Species pool size × Immigration) × Year	0.01	0.02	-0.04 – 0.05	0.24	0.807	2590
**Random Effects**						
σ^2	0.02					
$\tau_{00\ site}$	0.00					
$\tau_{11\ site.year}$	0.00					
$\rho_{01\ site}$	0.57					
ICC	0.08					
N_{site}	12					
Observations	2602					
Marginal R^2 / Conditional R^2	0.11 / 0.18					

Table S3. Local α-diversity linear mixed model results fit using Restricted Maximum Likelihood and Satterthwaite degrees of freedom. α-diversity was natural log transformed to linearize relationships and satisfy model assumptions. Input variables Immigration and Year were standardized by centering and dividing by two standard deviations prior to fitting model.

Predictors	*Estimates*	*std. Error*	*CI*	*Statistic*	*p*	*df*
(Intercept)	0.47	0.05	0.37 – 0.58	8.73	<0.001	2586
Species pool size	0.25	0.02	0.21 – 0.28	15.92	<0.001	2586
Immigration	0.11	0.02	0.07 – 0.16	5.17	<0.001	2586
Year [1st degree]	-3.55	1.67	-6.82 – -0.28	-2.13	0.033	2586
Year [2nd degree]	-2.20	0.55	-3.29 – -1.11	-3.97	<0.001	2586
Species pool size × Immigration	0.13	0.03	0.07 – 0.19	4.24	<0.001	2586
Species pool size × Year [1st degree]	-0.37	0.79	-1.91 – 1.17	-0.47	0.637	2586
Species pool size × Year [2nd degree]	-0.16	0.79	-1.70 – 1.38	-0.20	0.841	2586
Immigration × Year [1st degree]	-2.82	1.12	-5.01 – -0.63	-2.52	0.012	2586
Immigration × Year [2nd degree]	-1.19	1.12	-3.38 – 1.01	-1.06	0.289	2586
(Species pool size × Immigration) × Year [1st degree]	-1.38	1.57	-4.46 – 1.71	-0.88	0.381	2586
(Species pool size × Immigration) × Year [2nd degree]	1.91	1.57	-1.17 – 5.00	1.22	0.224	2586
**Random Effects**						
σ^2	0.15					
$\tau_{00\ site}$	0.03					
$\tau_{11\ site.year}$	0.05					
$\rho_{01\ site}$	-0.06					
ICC	0.18					
N_{site}	12					
Observations	2602					
Marginal R^2 / Conditional R^2	0.15 / 0.30					

Table S4. Effects of Immigration, species pool size, and time (year) on the amount of variation in community composition explained by landscape-scale gradient in soil resources, quantified with distance-based redundancy analysis. Variation in community composition was calculated as total Bray-Curtis dissimilarity (β-diversity) and its abundance-based components: balanced variation in abundance (β_{bal}) and gradients in abundance (β_{gra}). Input variables Immigration and Year were standardized by centering and dividing by two standard deviations prior to fitting log-normal multiple regression models.

Model	*Predictors*	*Estimates*	*std. Error*	*CI*	*Statistic*	*p*
β-diversity						
	(Intercept)	-2.85	0.09	-3.04 – -2.66	-30.24	<0.001
	Immigration	0.31	0.14	0.04 – 0.59	2.31	0.027
	Species pool size	-0.29	0.13	-0.56 – -0.02	-2.19	0.035
	Year	0.76	0.14	0.48 – 1.03	5.61	<0.001
β_{bal}						
	(Intercept)	-2.07	0.11	-2.30 – -1.84	-18.34	<0.001
	Immigration	0.31	0.16	-0.02 – 0.63	1.91	0.065
	Species pool size	-0.43	0.16	-0.75 – -0.10	-2.69	0.011
	Year	0.89	0.16	0.56 – 1.21	5.49	<0.001
β_{gra}						
	(Intercept)	-3.26	0.11	-3.49 – -3.03	-28.55	<0.001
	Immigration	0.14	0.16	-0.20 – 0.47	0.83	0.411
	Species pool size	0.2	0.16	-0.12 – 0.53	1.26	0.216
	Year	-0.06	0.16	-0.39 – 0.27	-0.36	0.719

Table S5. Effects of immigration, species pool size, and time (year) on the probability the dominant species A. gerardii occupied a plot. Odds ratios were estimated from a generalized linear mixed model specifying the binomial distribution and logit link (e.g., logistic model) with a random intercept for “site”. The immigration predictor was standardized prior to model fitting. See model predictions in Fig. S9.

Predictors	*Odds Ratios*	*std. Error*	*CI*	*Statistic*	*p*
(Intercept)	0.22	0.07	0.12 – 0.41	-4.86	**<0.001**
Pool [Large]	0.47	0.05	0.38 – 0.58	-6.99	**<0.001**
scaled immigration	1.28	0.07	1.15 – 1.42	4.63	**<0.001**
Year	2.74	0.13	2.50 – 3.00	21.87	**<0.001**
**Random Effects**					
σ^2	3.29				
$\tau_{00\ site}$	0.95				
ICC	0.22				
N_{site}	12				
Observations	2602				
Marginal R^2 / Conditional R^2	0.342 / 0.489				

Table S6. Effects of immigration and species pool size on initial community size (total number of individual seedlings in the first year following seed addition). Immigration (seed density) was standardized, and first year seedling counts were natural-log transformed prior to fitting models as described for diversity responses. See model predictions in Fig. S2.

Predictors	*Estimates*	*std. Error*	*CI*	*Statistic*	*p*	*df*
(Intercept)	3.68	0.20	3.28 – 4.08	18.21	**<0.001**	283.00
Species pool [Large]	-1.02	0.08	-1.17 – -0.86	-12.61	**<0.001**	283.00
Immigration	0.92	0.08	0.76 – 1.08	11.35	**<0.001**	283.00
**Random Effects**						
σ^2	0.47					
$\tau_{00\ site}$	0.23					
ICC	0.33					
N_{site}	6					
Observations	288					
Marginal R^2 / Conditional R^2	0.404 / 0.598					
